# Regulation of Transcription Elongation and Termination

**DOI:** 10.3390/biom5021063

**Published:** 2015-05-29

**Authors:** Robert S. Washburn, Max E. Gottesman

**Affiliations:** 1 Department of Microbiology and Immunology, Columbia University Medical Center, New York, NY 10032, USA; E-Mail: rw378@cumc.columbia.edu; 2 Department of Biochemistry and Molecular Biophysics, Columbia University Medical Center, New York, NY 10032, USA

**Keywords:** transcription elongation, transcription pausing, transcription termination, Nus factors, transcription antitermination, transcription-translation coupling

## Abstract

This article will review our current understanding of transcription elongation and termination in *E. coli*. We discuss why transcription elongation complexes pause at certain template sites and how auxiliary host and phage transcription factors affect elongation and termination. The connection between translation and transcription elongation is described. Finally we present an overview indicating where progress has been made and where it has not.

## 1. Introduction

This article will review our current understanding of transcription elongation and termination in *E. coli.* Because of the large scope of the article, we have largely confined ourselves to recent manuscripts. Older references have, in general, been omitted except when necessary or when they consist of our own work.

RNA synthesis by bacterial RNA polymerases, although processive, does not proceed at a uniform rate. Template sequences can induce pausing or backtracking—movement of the transcription elongation complex (TEC) towards the promoter. Forward or backward movement of TEC does not entail loss of energy. The RNA:DNA hybrid, 9 nt in the post-translocated state and 10 nt in the pre-translocated state, is maintained independently of the direction of RNAP movement.

Translocation speed and direction is also influenced by accessory transcription factors. NusG and NusA suppress or enhance pausing, respectively. UvrD can push TEC backwards to reveal damaged DNA, and Mfd can push it forward as part of the transcription-coupled repair process. The GreA/B proteins can rescue backtracked TEC by removing extruded RNA and restoring the 3'- end of the nascent transcript to the TEC active center. Backtracking is also suppressed when TEC is coupled to translating ribosomes, which present a barrier to retrograde translocation. Phage functions affect transcription elongation. λ N protein accelerates transcription by inhibiting pausing, whereas HK022 arrests transcription by preventing TEC translocation.

Transcription terminates at the ends of operons or under certain conditions, within genes. Termination can be template-encoded and factor-independent (intrinsic termination), or require accessory factors, such as Rho, Mfd and DksA.

There has been considerable progress in understanding these aspects of transcription elongation, although areas of controversy remain. We will summarize the new findings and try to smooth out the contradictions in the following pages.

## 2. Pausing

The rate of transcription elongation by *E. coli* RNAP is not uniform. RNA synthesis is characterized by pauses, some of which may be brief and resolved spontaneously, whereas others may lead to TEC backtracking. Backtracked TEC can restart when acted upon by GreA/B factors, which restore the 3'- end of the nascent transcript to the active center. Pausing may regulate gene expression, as in the case of attenuation or phage λ gene Q antitermination. In this section we will discuss recent advances in understanding the mechanism and specificity of pausing.

Elongation rate and pausing are determined by template sequence and RNA structure (e.g., stem-loops) and involve at least two components of the RNAP catalytic center, the bridge helix (BH) and trigger loop (TL). Elongation is proposed to occur in two steps. First, the TL folds in response to NTP binding. Mutational analyses indicate that this conformational change in the TL can be rate-limiting, and reflects the ability of the incoming NTP to bind to TEC. The second step is the incorporation of the NTP and the release of pyrophosphate. Kinetic conformational changes in the TL, however, do not account for pause recovery, since the TL remains unfolded during a pause [1].

Pausing not associated with backtracking is frequent, occurring on average every 100 bases of DNA [2]. The paused intermediates are distinct from the intermediates of the main reaction pathway, and they are not associated with translocation delay. The paused complex contains the 3'- end of the transcript in the active center and is capable of binding the next cognate NTP. It is highly dependent on the NMP at the 3'OH end of the nascent transcript. For example, pausing at C37 on a T7A1 template is significantly reduced when the template substitutes a G37 for C37. Substitutions at position 38 also affect pause times, which might indicate the ability of the incoming XTP to bind to TEC. These considerations have lead to the idea that the 3'OH base may not be fully base-paired with the template, even though it lies in the post-translocated state.

Zenkin and his coworkers [3] analyzed pauses that result from failure of TEC to translocate from the elongation-inactive pre-translocated state to the active post-translocated state. These pauses reflect the ability of RNAP core to sense the identity of base pairs at most of the positions of the RNA–DNA hybrid. It is not clear if the sequence or the structure of the hybrid induces pausing. Some of these pauses are associated with “backstepping”, *i.e*., movement of TEC towards the promoter by one bp, with associated displacement of the 3' ribonucleotide from the active center.

A genome-wide *in vivo* analysis of TEC occupancy on the *E. coli* chromosome defined almost 20,000 pause sites [4]. Analysis of these sites revealed a consensus sequence that consists of G–10 Y–1 G+1 (where –1 corresponds to the position of the RNA 3' end). A similar result was obtained by Larson *et al*. [5] This sequence is proposed to induce pausing through an interaction between RNAP core enzyme and a core recognition element (CRE) located at the 3'- end of the RNA:DNA duplex. The interaction stabilizes TEC in the pre-translocated state, thus inhibiting addition of the next nucleotide to the nascent transcript. The G-10 favors the pretranslocated state by enhancing duplex stability; each position of the consensus pause sequence is predicted to favor the pretranslocated state over the posttranslocated state (the –10G through effects on duplex stability, the –1 Y through effects on active-center binding, and the +1 G through both). Mutational probing of RNAP supports this model. Thus, RNAP βD446 hydrogen bonds with Watson-Crick atoms of G complexed with CRE, suggesting that D446 recognizes this nucleotide. As predicted, RNAP βD446A cannot distinguish G, A, T, or an abasic site at position G+1 *in vitro* and pauses with equal efficiency on the various templates. However, the mutant RNAP is more likely than wild-type enzyme to be in the pretranslocated register on the G+1 template, *i.e*., more likely to pause. This observation is curious, and does not fit readily with the above model.

Interestingly, the pause-inducing consensus sequence is enriched at translation start sites in both *E. coli* and *Bacillus subtilis* [5]. It is conceivable that these pause sites play a regulatory role coupling transcription and translation by allowing linkage of the lead ribosome to RNAP.

Pauses also occur at sites resembling the promoter sequence to which the RNAP sigma 70 subunit binds [6,7]. Sigma 70 engages the promoter-like sequence and TEC, which briefly continues RNA synthesis. Sigma-dependent pausing generates stressed elongation complexes that are resistant to GreA and GreB cleavage, suggesting that the 3'- end of the RNA is in the active site, as would be expected in a paused, scrunched complex. The scrunched complexes are resolved either by breakage of the TEC—sigma 70—promoter-like sequence, or by isomerization to a backtracked conformation. TEC involved in this type of pause may consist of persistent holoenzymes, in which σ70 regions 1.2 and 2 remain in contact with the RNAP core. The pausing frequency of elongating holoenyzme is not clear, and may depend on the growth rate of the bacteria [8].

## 3. Intrinsic Termination

**Figure 1:**
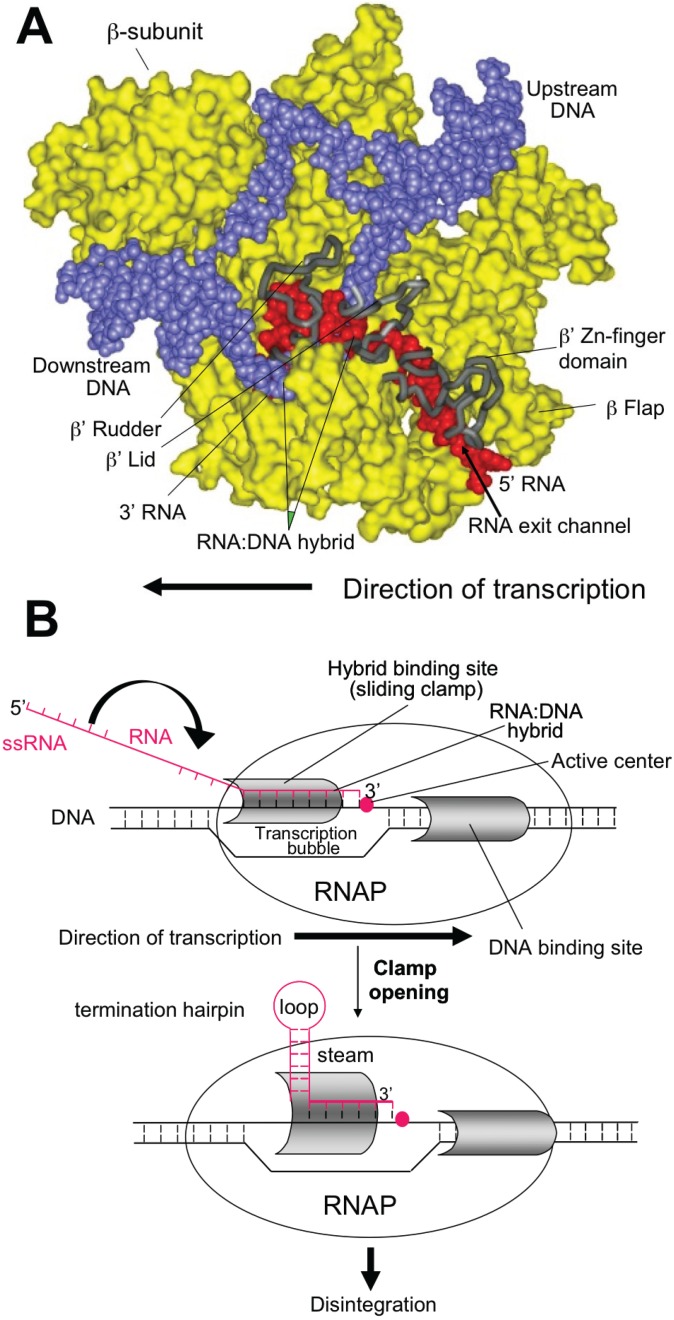
Model of Intrinsic termination.

Intrinsic termination occurs at specific template sequences - an inverted repeat followed by a run of A residues. Termination is driven by formation of a short stem-loop structure in the nascent RNA chain. RNA synthesis arrests and TEC dissociates at the 7th and 8th U of the run. Formation of the stem-loop dissociates the weak rU:dA hybrid. Stem-loop formation is hindered by upstream complementary RNA sequences that compete with the downstream portion of the stem, as well as by RNA: protein interactions in the RNA exit channel. Intrinsic termination depends critically upon timing. Hairpin folding and transcription of the termination point must be coordinated, so that the complete hairpin is formed by the time RNAP transcribes the termination point. The size of the stem, the sequence of the stem and the length of the loop all affect termination efficiency (see Figure 1).

Nedialkov *et al*. [9] have studied the role of RNAP domains in intrinsic termination. The bridge α-helix in the β' subunit borders the active site and may have roles in catalysis and translocation. Mutations in the YFI motif (β' 772-YFI-774) affect intrinsic termination as well as pausing, fidelity and translocation of RNAP. One mutation, F773V, abolishes the activity of the λ tR2 intrinsic terminator, although neighboring mutations have little affect on termination. Modeling suggests that this unique phenotype reflects the ability of F773 to interact with the fork domain in the β subunit.

## 4. Rho-Dependent Termination

Transcription termination factor Rho is an essential protein in *E. coli* first identified for its role in transcription termination at Rho-dependent terminators [10], and is estimated to terminate ~20% of *E. coli* transcripts [11]. *rho* is highly conserved and nearly ubiquitous in bacteria [12]. Rho is an RNA-dependent ATPase [13] with RNA:DNA helicase activity [14], and consists of a hexamer of six identical monomers arranged in an open circle [15]. Transcription of the single copy of *rho* is regulated by Rho-dependent transcription termination at a Rho termination site located upstream of the structural gene [16].

Rho-dependent terminators are comprised of *rut* sites (*r*ho *ut*ilization) and release sites [17]. Rho binds with a high affinity to the *rut* site RNA, is stimulated to hydrolyze ATP, and then translocates along the RNA in a 5' to 3' direction while maintaining an interaction with *rut* [18,19], until it encounters TEC at a release site. Termination requires untranslated RNA of at least 85–97nt [20].

**Figure 2:**
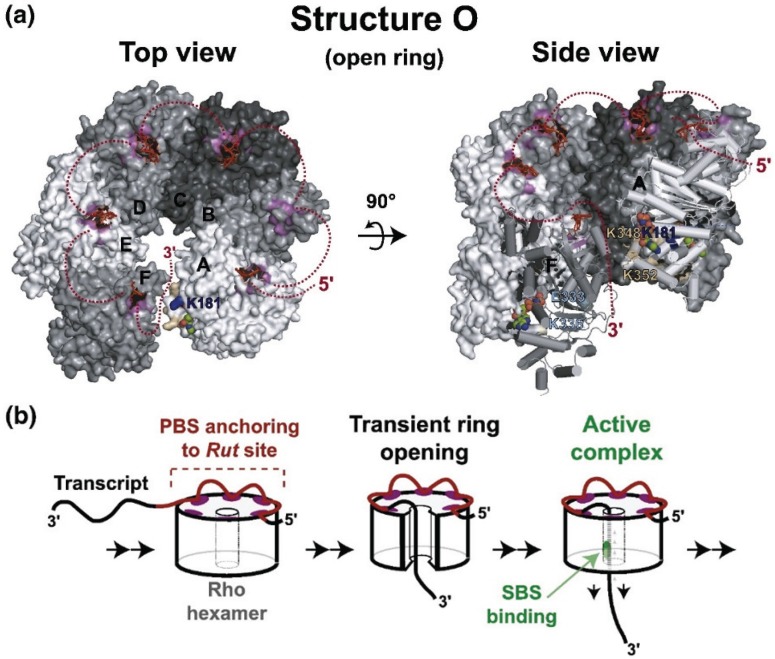
Structure and movement of Rho.

RNA binds to two distinct sites in Rho, termed primary and secondary [21]. The primary site stably binds RNA in the absence of ATP. The secondary sites are stimulated to bind RNA transiently after the primary site is occupied. Secondary site binding stimulates ATP hydrolysis. Crystallography has identified the location of the primary site on the outer edge of the hexamer and the secondary site around the center hole [15,22]. The Rho hexamer initially binds RNA in an open “lockwasher” conformation. After RNA is bound to the primary site, the transcript is threaded through the central hole contacting the secondary binding site and the hexamer closes (Figure 2, [23]). Single molecule experiments determined that Rho binds 57 ± 2 nucleotides of RNA in the absence of ATP and 80 ± 2 nucleotides upon ATP hydrolysis, consistent with 60 nt binding at the primary and 20 nt at the secondary site [19]. The transient interactions of RNA with the secondary site drive Rho translocation along the RNA until it encounters TEC at a release site. At this point, Rho releases RNAP from the template, presumably by unwinding the RNA-DNA hybrid. The precise mechanism of arrest and removal is unclear. Epshtein *et al*. [24] propose that Rho causes a conformational change in RNAP leading to arrest of TEC and exposure of the transcription hybrid to Rho. In this model, Rho then unwinds the RNA-DNA hybrid, removing RNAP. The authors argue that since crosslinking data indicates that the active site does not move in relation to the template during transcription termination, forward translocation does not contribute to termination. Park and Roberts [25], however, found that Rho induces forward movement of TEC, and that mispairings in the DNA template immediately 5' to the arrested TEC decrease the efficiency of Rho termination. Park and Roberts propose that Rho induces termination by pushing RNAP ahead of the transcription hybrid. The precise mechanism of removal is thus still unclear, but the above studies suggest that forward pressure on RNAP from Rho causes a conformational change leading to arrest. Continued pressure then exposes the transcription hybrid to Rho either by a conformational change in RNAP or by removal without forward translocation. Termination may not entail specific interactions with RNAP, since *E. coli* Rho factor will efficiently terminate transcription of *Saccharomyces cerevisiae* RNA pol II [26].

Rho-dependent terminators, unlike intrinsic terminators, lack an easily identifiable motif. *rut* sites are likewise not highly conserved. They consist of unstructured RNA that is C-rich and G-poor compared to the flanking sequences [20,27,28]. Cytidine residues most strongly activate the ATPase activity [13]. Release sites correlate with TEC pausing, but not all pause sites can function as efficient release sites [24,29]. Efficient Rho-dependent termination *in vivo* requires the NusG transcription factor [30]. NusG binds to Rho [31] and shifts termination to more 5' release sites [32,33,34]. Rho binds NusG weakly in solution (k_D_ = 0.2 μM; [31]) but NusG enhances Rho binding to RNAP *in vitro* [35].

ChIP-chip analysis reveals that Rho associates with the TEC throughout transcription, rather than only after synthesis of an untranslated *rut* binding site [36]. Kalyani *et al*. [37] instead maintain that a transcribed *rut* element is required for Rho association with RNAP, and suggest that the ChIP-chip data does not reflect a relevant interaction between Rho and TEC. Single molecule studies show no evidence that Rho directly binds to RNAP [19], however, Epshtein *et al*. [24] did detect direct binding to RNAP *in vitro*. The reason for the above discrepancy remains unclear.

Rho-dependent termination in *E. coli* occurs predominantly within the “foreign” DNA (e.g., cryptic prophages and transposons) that makes up ~14% of the genome of *E. coli MG1655* [38]. Efficient transcription termination in *E. coli* is essential to suppress expression of toxic genes in this horizontally-acquired DNA [38]. Although Rho activity can be significantly reduced by *nusA* or *nusG* mutations in a strain deleted of all horizontally transferred DNA, *rho* cannot be deleted in this strain. Washburn and Gottesman proposed that Rho is essential to prevent collisions between TEC and the replisome [39]. Rho termination within coding sequences may depend upon ribosome release by tmRNA, which can uncouple the lead ribosome from RNAP [40].

The RNA-DNA helicase activity of Rho can unwind extensive RNA-DNA hybrids. Harinarayanan and Gowrishankar [41] suggest that Rho prevents RNA-DNA hybrids (“R-loops”) from forming between untranslated mRNA and the chromosome. In support of this hypothesis, Leela *et al*. [42] found that *rho* could be deleted in a *rac^−^* strain expressing the RNA-DNA helicase *uvsW*. This model assumes that both Rho and UvsW prevent or remove potentially lethal R-loops. Nevertheless, *rnhA* deletion mutants are healthy despite extensive accumulation of R-loops. Nor are *rnhA* mutants more dependent on *rho* for survival than wild-type [43,44]. Perhaps Rho and UvsW share another essential activity, such as resolving conflicts between transcription and replication, or removing certain toxic R-loops sequestered from RNaseHI. Note that Dutta *et al*. [45], demonstrated that suppression of R-loop formation reduces transcription-replication clashes.

Peters *et al*. [46] found that inhibiting Rho or deleting *nusG* resulted in the accumulation of antisense transcripts. The antisense transcripts are untranslated, thus allowing Rho access to termination sites. Surprisingly, *nusA* does not contribute to termination of the antisense transcripts, despite the similarities in transcription patterns in *nusG*, *nusA* and cells treated with the Rho inhibitor bicyclomycin [38]. Whether or not this activity of Rho is important *in vivo* is unclear.

The efficiency of Rho termination is dependent on the rate of transcription elongation [47]. Slow RNAP mutants have a decreased affinity for NTPs (e.g., *rpoB8*, 5-fold higher Km for ATP) and are more efficient at Rho-dependent termination. Conversely, fast mutants have an increased affinity for NTPs (e.g., *rpoB3595*) and are less efficiently terminated. Enhanced termination was observed *in vitro* when the transcription rate was slowed by limiting NTP concentrations. This “kinetic coupling” model might explain the correlation between TEC pausing and Rho termination sites.

The histone-like nucleoid-structuring protein H-NS contributes to Rho-dependent termination. *hns* deletion mutations increase Rho dependency [43,44], and H-NS is concentrated at the antisense transcription terminators [46]. This is distinct from the role of H-NS in silencing foreign genes by repressing promoters [48]. Horizontally-transferred DNA is AT rich compared to *E. coli* sequences, which favors H-NS binding.

## 5. NusG, NusA and DksA

TEC are accompanied by transcriptional cofactors that affect the rate of elongation and specify loci of transcription termination. Among these are NusA, NusG, and the ppGpp cofactor, DksA.

### 5.1. NusG

The 21kDa *E. coli* NusG is composed of two domains connected by a flexible linker. It affects transcription elongation through a variety of mechanisms. The NusG-NTD directly suppresses pausing and thus enhances the overall rate of transcription elongation [49]. Structural studies with the archaeal NusG homologue, Spt5, suggests that the NusG-NTD enhances TEC processivity by completely encircling the DNA binding channel of RNAP, thus stabilizing the closed conformation of the RNAP clamp domain [50]. Single molecule analyses indicate that NusG decreases the rate of entry into both short-lifetime and, more significantly, long-lifetime pauses. Suppression of long-lifetime pauses is proposed to account for NusG-NTD enhancement of transcription elongation. According to this model, NusG-NTD increases movement of TEC along the DNA template by promoting transition from the pre-translocated towards the post-translocated register [51].

The NusG-CTD KOW domain interacts with NusE/S10, thus linking TEC to the lead ribosome. Coupling of transcription to translation suppresses backtracking and possible clashes with the replisome [45]. The NusG-CTD also binds to—and activates—termination factor Rho with the same interface with which it binds NusE/S10. Thus ribosome-associated NusG-CTD is not available to enhance Rho-dependent termination [52]. Linkage between the lead ribosome and TEC also suppresses formation of untranslated RNA, which is required for Rho to access TEC. Sequestering of the NusG-CTD and the absence of RNA ligand together account for the absence of Rho-dependent termination in well-translated genes.

*In vivo*, it is not known whether NusG associates first with RNAP, with ribosomes or simultaneously to both. Genome-wide surveys suggest that NusG associates with TEC only after significant elongation has occurred [36]. This is difficult to reconcile with the coupling hypothesis, since free TEC could be targeted by Rho (see below). On the other hand, the *E. coli* NusG paralogue, RfaH, appears to link ribosomes to TEC early after transcription initiation [53].

The *in vivo* calculated/reported numbers for NusG [54] is one sixth the number of ribosomes (≈55,000 copies/cell, [55]). This is consistent with the idea that only the first ribosome in translating polysomes associates with NusG and TEC.

However, ribosomal stalling at rare codons or induced by amino acid analogues can uncouple transcription from translation and induce intragenic Rho-dependent termination. The stalled ribosome is attacked by tmRNA, which competes for binding with NusG-CTD to S10 and releases the impacted ribosome [40].

Activation of Rho explains why NusG is essential in wild-type *E. coli*. The cryptic *rac* prophage carries a constitutive promoter and a downstream *kil* gene whose expression is lethal to the bacterial host. Rho-dependent termination prevents transcription extension from the promoter to *kil*. Deletion of the *rac* prophage allows *E. coli* to support a *nusG* deletion, although the mutant strain grows poorly and dies in stationary phase [38].

Oddly, *B. subtilis* NusG stimulates pausing at two regulatory sites in the untranslated leader of the *B. subtilis trp* operon that participate in transcription attenuation and translational control mechanisms, respectively. To induce pausing, *B. subtilis* NusG makes sequence-specific contacts with a T-rich sequence in the nontemplate DNA strand within the paused transcription bubble [56]. The *E. coli* NusG homologue, RfaH, makes similar contacts with the template *ops* element. Pausing at *ops* may allow RfaH to link to the lead ribosome and couple transcription to translation in *ops*-bearing operons [57].

### 5.2. NusA

*E. coli* NusA protein was originally identified genetically as a required component of the phage λ N antitermination complex, and biochemically as a factor that stimulated *lacZ* gene expression *in vitro* [58]. RNAP is modulated by NusA protein and *vice versa*. NusA enhances pausing as well as termination at intrinsic termination sites. Paradoxically, it also suppresses transcription termination as part of the λ N or *rrn* antitermination complexes. NusA is thought to provoke termination when present in 1:1 stochiometry with RNAP, and antitermination—as part of the NusBEG/λ N complex—when the stoichiometry is 2:1. Numerous studies place NusA near the RNA exit channel [59]. Gusarov and Nudler [60] proposed that NusA weakens RNA binding to the upstream bindings elements (UBS) in the channel, allowing formation of the RNA stem-loop that induces termination at intrinsic terminators. However, direct binding of NusA-NTD to RNA:RNA duplexes in the exit channel has recently been demonstrated [61]. Thus, direct stabilization of RNA:RNA hybrids in the exit channel might instead be responsible for NusA stimulation of pausing and intrinsic termination.

The NusABEG/λN antitermination complex forms at the NUT sequences of λ nascent transcript. These sequences lie in the phage chromosome between the λ*pL* and λ *pR* promoters and the first termination signals in their operons. NusA binds to the NUT SPACER sequence within NUT. However, NusA binding to RNA is dependent on its association with TEC. NusA binds to TEC via two distinct domains, the NTD and an acidic domain in the CTD (AR2). The C-terminal domain (CTD) of the RNAP α-subunit (αCTD) interacts with the acidic CTD 2 (AR2) of NusA, releasing the autoinhibitory blockade of the NusA S1-KH1-KH2 motif and allowing NusA to bind RNA. The solution conformation of the AR2:αCTD complex shows that the αCTD residues that interface with AR2 are identical to those that recognize UP promoter elements. This is consistent with a role for NusA in transcription initiation of operons carrying UP elements, although evidence for such an activity has yet to be unearthed [62].

Earlier studies suggested that the binding of NusA-NTD and σ70 to RNAP were mutually exclusive. However, recent structural data shows association of NusA-NTD and the β-flap tip helix, a site distinct from that of the major σ70 region 2 binding site, the CH region of the β' subunit. Nevertheless, NusA might compete with the weak binding of σ70 region 4 to the β-flap tip [63].

Unlike NusG, complete deletions of NusA cannot be constructed. A NusA truncation that retains the NusA-NTD can be introduced into strain MDS42, which lacks all horizontally transmitted elements, including the cryptic *rac* prophage [64]. This is explained by the finding that the NusA-NTD has biochemical activity; NusA-NTD by itself is necessary and sufficient for enhancement of transcriptional pausing. The other, dispensable, NusA domains provide additional, interactions with TEC and are required to stimulate intrinsic termination [61].

### 5.3. DksA

DksA was originally isolated as a suppressor of a chaperone mutation, *dnaK*. It was then shown to disrupt RNAP open complexes at *rrn* promoters under the influence of ppGpp and NTP [65]. DksA and GreA/B belong to a family of coiled-coil proteins that bind within the secondary channel of RNAP. Despite structural similarities to GreA/B, DksA cannot induce RNAP to cleave RNA in backtracked RNAP.

The critical difference between DksA and GreA/B lies in a few residues at the tip of the coiled coil. These residues contact the RNAP active center [66]. There is physiological interplay between the two functions. Thus, microarray analysis indicates that many genes are similarly regulated by DksA and GreA. GreA overproduction can suppress a *dksA* growth defect. At other genetic loci, however, DksA and GreA act oppositely. The biochemical basis of these interactions remains to be elucidated [67]. Like DksA, GreA can act at promoters, where it facilitates promoter escape. In particular, this activity of GreA strongly stimulates expression of ribosomal protein operons and the *tna* operon [68].

The fact that ppGpp inhibits RNA chain extension prompted experiments to test if DksA also acted on TEC *in vitro*. Although wild-type DksA has little or no effect on the rate of RNA synthesis with wild-type RNAP, a DksA mutant with enhanced affinity for RNAP slows elongation in a ppGpp-independent fashion, although this effect is stimulated by ppGpp. Similarly, wild-type DksA retards RNA synthesis by an RNAP mutant with increased sensitivity to DksA, again independently of ppGpp. The template used in these studies lacked paused sites, suggesting that DksA does not slow transcription elongation by stimulating pausing. Finally, DksA stimulates termination at the intrinsic *rrnB* T1 terminator [69].

*In vivo*, mutational studies implicate DksA in preventing transcription-replication conflicts. DksA protects cells against UV and other DNA damage, which inhibit transcription elongation [70]. DksA prevents replication arrest in amino acid-starved cells via effects on transcription elongation [71]. Amino acid starvation, which stalls translation, arrests DNA replication in the absence of DksA [71]. This is consistent with the idea that TEC can uncouple from stalled ribosomes and, if not removed by Rho, will backtrack and form a barrier to replication [39,64]. Tehranchi *et al*. [71] propose that DksA prevents backtracking of uncoupled TEC (rather than resolving backtracked TEC), and, therefore, suppresses replisome clashes. The mechanism by which DksA might accomplish this reaction remains undefined.

A genome wide survey of TEC occupancy in the presence or absence of DksA supports the notion that DksA suppresses replisome clashes by acting on backtracked TEC. ChIP-chip experiments reveal that DksA is enriched both at promoters and in downstream regions, colocalizing with RNAP across the entire chromosome. DksA suppresses TEC stalling induced by amino acid starvation globally, possibly by blocking backtracking [72]. An alternate interpretation, that DksA removes uncoupled TEC ahead of the replisome, suggested by *in vitro* studies, has not been ruled out [69]. Genetic evidence further complicates the picture. Thus the sensitivity of *dksA* mutants to the DNA cross-linker, mitomycin C, is suppressed by a second mutation in *greA* [73]. Since both mutations are proposed to increase backtracking or stabilize the backtracked TEC, it is difficult to rationalize this suppression pattern. Reconciling these *in vitro* and *in vivo* observations will be, we are afraid, the task of future generations.

## 6. RNA-Binding Phage-Encoded Proteins that Affect Transcription Elongation

### 6.1. λ N Antitermination

λ N suppresses transcription termination *in vivo* specifically on the λ chromosome. It is directed to TEC by binding to the NUT sequence of the λ nascent transcript via its N-terminal arginine rich motif (ARM), and remains attached to TEC during transcription of the λ early genes. λ N and NusA, B, E and G form an antitermination complex that modifies TEC. The mechanism of action of λ N remains controversial. *In vitro*, λ N alone can accelerate transcription elongation and suppress transcription termination at Rho-dependent and intrinsic termination sites. However, *E. coli* NusA factor significantly improves λ N efficiency. Gusarov and Nudler [60] found that λ N has no effect on RNA:DNA hybrid stability in TEC, with or without NusA, suggesting that these factors do not suppress hairpin formation and intrinsic termination by strengthening the hybrid. However, Parks *et al*. [74] concluded that λ N protein reduces transcriptional slippage within actively growing cells and *in vitro*. This result suggests that λ N does, in fact, stabilize the RNA:DNA hybrid, particularly at the 5' end. Stabilization is proposed to disfavor dissociation of RNA from the DNA template, thereby suppressing both termination and slippage. In contrast, Gusarov and Nudler [60] suggest that λ N blocks hairpin formation by sequestering the ascending portion of the RNA stem, prohibiting annealing with the descending portion. Clearly, how λ N modifies transcription elongation remains an open question.

### 6.2. HK022 Nun-Mediated Transcription Arrest

HK022 blocks the growth of phage λ by arresting transcription at pause sites distal to the λ *nut* elements. The arrested TEC is released by the host Mfd factor, thus prematurely terminating transcription on the λ chromosome [75]. Nun binds NUT RNA with its ARM motif, whereas the C-terminal region of Nun interacts with TEC. Other than the effect of Nun on λ growth, no other biological function has been described for the protein. Lytic growth of HK022 is unaffected by *nun* mutations, and HK022 *nun* mutants lysogenize with normal frequency. The specificity of Nun exclusion is unique; other phage exclusion systems are active against a broad range of superinfecting phage [60].

It has been suggested that the function of the λ NUT RNA is to tether Nun or λ N, increasing the local concentration of the protein near TEC. Indeed, *nut* is dispensable for function of both proteins *in vitro* (see below; [74,76]). Furthermore, Nun overproduction is toxic to *E. coli*, although λ NUT sites are not encoded in the bacterial chromosome [77]. Toxicity is related to transcription termination, since host RNAP and Nun mutations that block Nun termination also suppress cell killing [77,78]. *In vivo*, Nun arrest requires the four *E. coli* auxiliary transcription elongation factors, NusA, NusB, NusE and NusG. Though these factors are not essential for Nun arrest *in vitro*, they enhance Nun specific activity, reducing the concentration of Nun required to block elongation [78].

**Figure 3:**
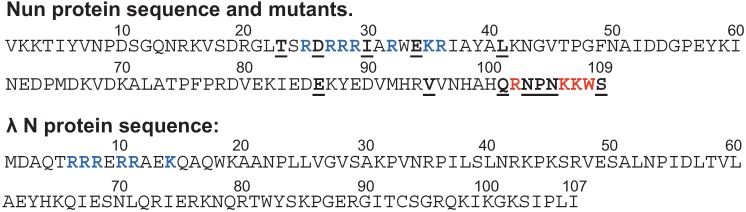
The sequence of Nun and N proteins. Blue—residues of ARM motif. Red—Residues with arrest-deficient phenotype. Underlined—other mutations affecting Nun activity.

TEC paused by nucleotide deprivation *in vitro* is a substrate for subsequent Nun arrest, although the site of arrest differs depending on the location of the pause [79,80]. Mutational analysis of the Nun C-terminus indicates that a penultimate aromatic residue (W108) is required for Nun arrest (Figure 3, top). The Nun C-terminus crosslinks to template DNA about 9 bp promoter-distal to the RNAP active center. This is compatible with the idea that Nun arrests transcription by anchoring TEC to the DNA template, perhaps via intercalation of the W108 residue. Two neighboring basic Nun C-terminal residues, K106 and K107 (Figure 3, top) are required for efficient arrest. They are thought to aid Nun binding to the negatively-charged DNA template. The Nun mechanism of action was tested on defined TEC scaffolds consisting of DNA template and non-template strands and RNA complementary to the template strand. These TECs differed in the length and the sequences of the RNA primer. Importantly, the scaffolds included no λ DNA or RNA sequences. Nun arrested all TECs tested that carried an RNA:DNA hybrid 9 bp or longer. For each TEC, Nun-mediated arrest occurred at a specific site, corresponding to an intrinsic pause site [77,81,82]. Nun-arrested TEC were found in either the pretranslocated or the posttranslocated state. Nun arrests transcription elongation by preventing movement of TEC from one register to the other [79,80].

## 7. Conclusions

Our understanding of transcription elongation has accelerated over the past few years. To a large extent, this reflects the application of structural biology to the elongation reaction, which, in turn, has informed the genetics, allowing construction of relevant mutant RNAPs and auxiliary factors. Nevertheless, we lack a satisfactory mechanistic explanation for the activities of many transcription factors, e.g., Rho, DksA, λ N and HK022 Nun remain obscure. This is likely to be so only in the short term, we expect, as more sophisticated structural and biochemical approaches are applied to determining how genes are transcribed.

## References

[1] Mejia Y.X., Nudler E., Bustamante C. (2015). Trigger loop folding determines transcription rate of *Escherichia coli*’s RNA polymerase. Proc. Natl. Acad. Sci. USA.

[2] Kireeva M.L., Kashlev M. (2009). Mechanism of sequence-specific pausing of bacterial RNA polymerase. Proc. Natl. Acad. Sci. USA.

[3] Bochkareva A., Yuzenkova Y., Tadigotla V.R., Zenkin N. (2012). Factor-independent transcription pausing caused by recognition of the RNA-DNA hybrid sequence. EMBO J..

[4] Vvedenskaya I.O., Vahedian-Movahed H., Bird J.G., Knoblauch J.G., Goldman S.R., Zhang Y., Ebright R.H., Nickels B.E. (2014). Transcription. Interactions between RNA polymerase and the “core recognition element” counteract pausing. Science.

[5] Larson M.H., Mooney R.A., Peters J.M., Windgassen T., Nayak D., Gross C.A., Block S.M., Greenleaf W.J., Landick R., Weissman J.S. (2014). A pause sequence enriched at translation start sites drives transcription dynamics *in vivo*. Science.

[6] Purdue S.A., Roberts J.W. (2011). σ^70^-dependent transcription pausing in *Escherichia coli*. J. Mol. Biol..

[7] Zhilina E., Esyunina D., Brodolin K., Kulbachinskiy A. (2012). Structural transitions in the transcription elongation complexes of bacterial RNA polymerase during σ-dependent pausing. Nucleic Acids Res..

[8] Wade J.T., Struhl K. (2004). Association of RNA polymerase with transcribed regions in *Escherichia coli*. Proc. Natl. Acad. Sci. USA.

[9] Nedialkov Y.A., Opron K., Assaf F., Artsimovitch I., Kireeva M.L., Kashlev M., Cukier R.I., Nudler E., Burton Z.F. (2013). The RNA polymerase bridge helix YFI motif in catalysis, fidelity and translocation. Biochim. Biophys. Acta.

[10] Roberts J.W. (1969). Termination factor for RNA synthesis. Nature.

[11] Peters J.M., Mooney R.A., Kuan P.F., Rowland J.L., Keles S., Landick R. (2009). Rho directs widespread termination of intragenic and stable RNA transcription. Proc. Natl. Acad. Sci. USA.

[12] Opperman T., Richardson J.P. (1994). Phylogenetic analysis of sequences from diverse bacteria with homology to the *Escherichia coli* Rho gene. J. Bacteriol..

[13] Lowery-Goldhammer C., Richardson J.P. (1974). An RNA-dependent nucleoside triphosphate phosphohydrolase (ATPase) associated with Rho termination factor. Proc. Natl. Acad. Sci. USA.

[14] Brennan C.A., Dombroski A.J., Platt T. (1987). Transcription termination factor Rho is an RNA-DNA helicase. Cell.

[15] Skordalakes E., Berger J.M. (2003). Structure of the Rho transcription terminator: Mechanism of mRNA recognition and helicase loading. Cell.

[16] Matsumoto Y., Shigesada K., Hirano M., Imai M. (1986). Autogenous regulation of the gene for transcription termination factor Rho in *Escherichia coli*: Localization and function of its attenuators. J. Bacteriol..

[17] Chen C.Y., Richardson J.P. (1987). Sequence elements essential for Rho-dependent transcription termination at λ tR1. J. Biol. Chem..

[18] Steinmetz E.J., Platt T. (1994). Evidence supporting a tethered tracking model for helicase activity of *Escherichia coli* Rho factor. Proc. Natl. Acad. Sci. USA.

[19] Koslover D.J., Fazal F.M., Mooney R.A., Landick R., Block S.M. (2012). Binding and translocation of termination factor Rho studied at the single-molecule level. J. Mol. Biol..

[20] Hart C.M., Roberts J.W. (1991). Rho-dependent transcription termination. Characterization of the requirement for cytidine in the nascent transcript. J. Biol. Chem..

[21] Richardson J.P. (1982). Activation of Rho protein ATPase requires simultaneous interaction at two kinds of nucleic acid-binding sites. J. Biol. Chem..

[22] Skordalakes E., Berger J.M. (2006). Structural insights into RNA-dependent ring closure and ATPase activation by the Rho termination factor. Cell.

[23] Rabhi M., Gocheva V., Jacquinot F., Lee A., Margeat E., Boudvillain M. (2011). Mutagenesis-based evidence for an asymmetric configuration of the ring-shaped transcription termination factor Rho. J. Mol. Biol..

[24] Epshtein V., Dutta D., Wade J., Nudler E. (2010). An allosteric mechanism of Rho-dependent transcription termination. Nature.

[25] Park J.S., Roberts J.W. (2006). Role of DNA bubble rewinding in enzymatic transcription termination. Proc. Natl. Acad. Sci. USA.

[26] Wu S.-Y., Platt T. (1993). Transcriptional arrest of yeast RNA polymerase II by *Escherichia coli* Rho protein *in vitro*. Proc. Natl. Acad. Sci. USA.

[27] Alifano P., Rivellini F., Limauro D. (1991). A consensus motif common to all Rho-dependent prokaryotic transcription terminators. Cell.

[28] Zalatan F., Platt T. (1992). Effects of decreased cytosine content on Rho interaction with the Rho-dependent terminator trp T' in *Escherichia coli*. J. Biol. Chem..

[29] Morgan W.D., Bear D.G., von Hippel P.H. (1983). Rho-dependent termination of transcription. I. Identification and characterization of termination sites for transcription from the bacteriophage λ PR promoter. J. Biol. Chem..

[30] Sullivan S.L., Gottesman M.E. (1992). Requirement for *E. coli* NusG protein in factor-dependent transcription termination. Cell.

[31] Li J., Mason S.W., Greenblatt J. (1993). Elongation factor NusG interacts with termination factor Rho to regulate termination and antitermination of transcription. Genes Dev..

[32] Burns C.M., Richardson L.V., Richardson J.P. (1998). Combinatorial effects of NusA and NusG on transcription elongation and Rho-dependent termination in *Escherichia coli*. J. Mol. Biol..

[33] Nehrke K.W., Zalatan F., Platt T. (1993). NusG alters Rho-dependent termination of transcription *in vitro* independent of kinetic coupling. Gene Expr..

[34] Washburn R.S., Jin D.J., Stitt B.L. (1996). The mechanism of early transcription termination by Rho026. J. Mol. Biol..

[35] Nehrke K.W., Platt T. (1994). A quaternary transcription termination complex. Reciprocal stabilization by Rho factor and NusG protein. J. Mol. Biol..

[36] Mooney R.A., Davis S.E., Mooney J.M., Rowland J.L., Ansari A.Z., Landick R. (2009). Regulator trafficking on bacterial transcription units *in vivo*. Mol. Cell..

[37] Kalyani B.S., Muteeb G., Qayyum M.Z., Sen J. (2011). Interaction with the nascent RNA is a prerequisite for the recruitment of Rho to the transcription elongation complex *in vitro*. J. Mol. Biol..

[38] Cardinale C.J., Washburn R.S., Tadigotla V.R., Brown L.M., Gottesman M.E., Nudler E. (2008). Termination factor Rho and its cofactors NusA and NusG silence foreign DNA in *E. coli*. Science.

[39] Washburn R.S., Gottesman M.E. (2011). Transcription termination maintains chromosome integrity. Proc. Natl. Acad. Sci. USA.

[40] Washburn R.S., Hashem Y., Sun M., Shen B., Harvey S., Frank J., Gottesman M.E. (2015). NusG and RelA couple and tmRNA uncouples transcription with translation in *E. coli*.

[41] Harinarayanan R., Gowrishankar J. (2003). Host factor titration by chromosomal R-loops as a mechanism for runaway plasmid replication in transcription termination-defective mutants of *Escherichia coli*. J. Mol. Biol..

[42] Leela J.K., Syeda A.H., Anupama K., Gowrishankar J. (2013). Rho-dependent transcription termination is essential to prevent excessive genome-wide R-loops in *Escherichia coli*. Proc. Natl. Acad. Sci. USA.

[43] Tran L., van Baarsel J.A., Washburn R.S., Gottesman M.E., Miller J.H. (2011). Single-gene deletion mutants of *Escherichia coli* with altered sensitivity to bicyclomycin, an inhibitor of transcription termination factor Rho. J. Bacteriol..

[44] Nichols R.J., Sen S., Choo Y.J., Beltrao P., Zietek M., Chaba R., Lee S., Kazmierczak K.M., Lee K.J., Wong A. (2011). Phenotypic landscape of a bacterial cell. Cell.

[45] Dutta D., Shatalin K., Epshtein V., Gottesman M.E., Nudler E. (2011). Linking RNA polymerase backtracking to genome instability in *E. coli*. Cell.

[46] Peters J.M., Mooney R.A., Grass J.A., Jessen E.D., Tran F., Landick R. (2012). Rho and NusG suppress pervasive antisense transcription in *Escherichia coli*. Genes Dev..

[47] Jin D.J., Burgess R.R., Richardson J.P., Gross C.A. (1992). Termination efficiency at Rho-dependent terminators depends on kinetic coupling between RNA polymerase and Rho. Proc. Natl. Acad. Sci. USA.

[48] Navarre W.W., Porwollik S., Wang Y., McClelland M., Rosen H., Libby S.J., Fang F.C. (2006). Selective silencing of foreign DNA with low GC content by the H-NS protein in Salmonella. Science.

[49] Mooney R.A., Schweimer K., Rösch P., Gottesman M., Landick R. (2009). Two structurally independent domains of *E. coli* NusG create regulatory plasticity via distinct interactions with RNA polymerase and regulators. J. Mol. Biol..

[50] Svetlov V., Nudler E. (2011). Clamping the clamp of RNA polymerase. EMBO J..

[51] Herbert K.M., Zhou J., Mooney R.A., la Porta A., Landick R., Block S.M. (2010). *E. coli* NusG inhibits backtracking and accelerates pause-free transcription by promoting forward translocation of RNA polymerase. J. Mol. Biol..

[52] Burmann B.M., Schweimer K., Luo X., Wahl M.C., Stitt B.L., Gottesman M.E., Rösch P. (2010). A NusE:NusG complex links transcription and translation. Science.

[53] Burmann B.M., Knauer S.H., Sevostyanova A., Schweimer K., Mooney R.A., Landick R., Artsimovitch I., Rösch P. (2012). An helix to barrel domain switch transforms the transcription factor RfaH into a translation factor. Cell.

[54] Torres M., Balada J.-M., Zellars M., Squires C., Squires C.L. (2004). *In vivo* effect of NusB and NusG on rRNA transcription antitermination. J. Bacteriol..

[55] Bakshi S., Siryaporn A., Goulian M., Weisshaar J.C. (2012). Superresolution imaging of ribosomes and RNA polymerase in live *Escherichia coli* cells. Mol. Microbiol..

[56] Yakhnin A.V., Babitzke P. (2014). NusG/Spt5: Are there common functions of this ubiquitous transcription elongation factor?. Curr. Opin. Microbiol..

[57] Tomar S.K., Knauer S.H., Nandymazumdar M., Rösch P., Artsimovitch I. (2013). Interdomain contacts control folding of transcription factor RfaH. Nucleic Acids Res..

[58] Nudler E., Gottesman M.E. (2002). Transcription termination and anti-termination in *E. coli*. Genes Cells.

[59] Kolb K.E., Hein P.P., Landick R. (2014). Antisense oligonucleotide-stimulated transcriptional pausing reveals RNA exit channel specificity of RNA polymerase and mechanistic contributions of NusA and RfaH. J. Biol. Chem..

[60] Gusarov I., Nudler E. (2001). Control of intrinsic transcription termination by N and NusA: The basic mechanisms. Cell.

[61] Ha K.S., Tiulokhonev I., Vassylyev D.G., Landick R. (2010). The NusA N-terminal domain is necessary and sufficient for enhancement of transcriptional pausing via interaction with the RNA exit channel of RNA polymerase. J. Mol. Biol..

[62] Schweimer K., Prasch S., Sujatha P.S., Bubunenko M., Gottesman M.E., Rösch P. (2011). NusA interaction with the α subunit of *E. coli* RNA polymerase is via the UP element site and releases autoinhibition. Structure.

[63] Yang X., Lewis P.J. (2010). The interaction between RNA polymerase and the elongation factor NusA. RNA Biol..

[64] Washburn R.S., Gottesman M.E. (2015).

[65] Ross W., Vrentas C.E., Sanchez-Vazquez P., Gaal T., Gourse R.L. (2013). The magic spot: A ppGpp binding site on *E. coli* RNA polymerase responsible for regulation of transcription initiation. Mol. Cell.

[66] Lee J.H., Lennon C.W., Ross W., Gourse R.L. (2012). Role of the coiled-coil tip of *Escherichia coli* DksA in promoter control. J. Mol. Biol..

[67] Vinella D., Potrykus K., Murphy H., Cashel M. (2012). Effects on growth by changes of the balance between GreA, GreB, and DksA suggest mutual competition and functional redundancy in *Escherichia coli*. J. Bacteriol..

[68] Stepanova E., Lee J., Ozerova M., Semenova E., Datsenko K., Wanner B.L., Severinov K., Borukhov S. (2007). Analysis of promoter targets for *Escherichia coli* transcription elongation factor GreA *in vivo* and *in vitro*. J. Bacteriol..

[69] Furman R., Sevostyanova A., Artsimovitch I. (2012). Transcription initiation factor DksA has diverse effects on RNA chain elongation. Nucleic Acids Res..

[70] Trautinger B.W., Jaktaji R.P., Rusakova E., Lloyd R.G. (2005). RNA polymerase modulators and DNA repair activities resolve conflicts between replication and transcription. Mol. Cell.

[71] Tehranchi A.K., Blankenschien M.D., Zhang Y., Halliday J.A., Srivatsan A., Peng J., Herman C., Wang J.D. (2010). The transcription factor DksA prevents conflicts between DNA replication and transcription machinery. Cell.

[72] Zhang Y., Mooney R.A., Grass J.A., Sivaramakrishnan P., Herman C., Landick R., Wang J.D. (2014). DksA guards elongating RNA polymerase against ribosome-stalling-induced arrest. Mol. Cell.

[73] Satory D., Halliday J.A., Sivaramakrishnan P., Lua R.C., Herman C. (2013). Characterization of a novel RNA polymerase mutant that alters DksA activity. J. Bacteriol..

[74] Parks A.R., Court C., Lubkowska L., Jin D.J., Kashlev M., Court D.L. (2014). Bacteriophage λ N protein inhibits transcription slippage by *Escherichia coli* RNA polymerase. Nucleic Acids Res..

[75] Washburn R.S., Wang Y., Gottesman M.E. (2003). Role of *E. coli* transcription-repair coupling factor Mfd in Nun-mediated transcription termination. J. Mol. Biol..

[76] Hung S.C., Gottesman M.E. (1995). Phage HK022 Nun protein arrests transcription on phage λ DNA *in vitro* and competes with the phage lambda N antitermination protein. J. Mol. Biol..

[77] Uc-Mass A., Khodursky A., Brown L., Gottesman M.E. (2008). Overexpression of phage HK022 Nun protein is toxic for *Escherichia coli*. J. Mol. Biol..

[78] Chattopadhyay S., Hung S.C., Stuart A.C., Palmer A.G., Garcia-Mena J., Das A., Gottesman M.E. (1995). Interaction between the phage HK022 Nun protein and the nut RNA of phage λ. Proc. Natl. Acad. Sci. USA.

[79] Vitiello C.L., Kireeva M.L., Lubkowska L., Kashlev M., Gottesman M. (2014). Coliphage HK022 Nun protein inhibits RNA polymerase translocation. Proc. Natl. Acad. Sci. USA.

[80] Vitiello C.L., Gottesman M.E. (2014). Bacteriophage HK022 Nun protein arrests transcription by blocking lateral mobility of RNA polymerase during transcription elongation. Bacteriophage.

[81] Rees W.A., Weitzel S.E., Yager T.D., Das A., von Hippel P.H. (1996). Bacteriophage λ N protein alone can induce transcription antitermination *in vitro*. Proc. Natl. Acad. Sci. USA.

[82] Watnick R.S., Gottesman M.E. (1998). *Escherichia coli* NusA is required for efficient RNA binding by phage HK022 nun protein. Proc. Natl. Acad. Sci. USA.

